# The mediating sex-specific effect of psychological distress on the relationship between adverse childhood experiences and current smoking among adults

**DOI:** 10.1186/1747-597X-7-30

**Published:** 2012-07-13

**Authors:** Tara W Strine, Valerie J Edwards, Shanta R Dube, Morton Wagenfeld, Satvinder Dhingra, Angela Witt Prehn, Sandra Rasmussen, Lela McKnight-Eily, Janet B Croft

**Affiliations:** 1 Office of Public Health Preparedness and Response, Office of Science and Public Health Practice, Centers for Disease Control and Prevention, Atlanta, GA, USA; 2 National Center for Chronic Disease Prevention and Health Promotion, Division of Adult and Community Health, Centers for Disease Control and Prevention, Atlanta, GA, USA; 3 National Center for Chronic Disease Prevention and Health Promotion, Office of Smoking and Health, Atlanta, GA, USA; 4 Walden University, Minneapolis, MN, USA; 5 Western Michigan University, Kalamazoo, MI, USA; 6 Williamsville Wellness, Hanover, VA, USA; 7 Office of Surveillance, Epidemiology, and Laboratory Services, Centers for Disease Control and Prevention, 2500 Century Pkwy Mailstop E-97, Atlanta, GA, 30345, USA

**Keywords:** Smoking, Adverse childhood experiences, Pyschological distress, Mediation analysis

## Abstract

****Background**:**

Research suggests that ACEs have a long-term impact on the behavioral, emotional, and cognitive development of children. These disruptions can lead to adoption of unhealthy coping behaviors throughout the lifespan. The present study sought to examine psychological distress as a potential mediator of sex-specific associations between adverse childhood experiences (ACEs) and adult smoking.

****Method**:**

Data from 7,210 Kaiser-Permanente members in San Diego California collected between April and October 1997 were used.

****Results**:**

Among women, psychological distress mediated a significant portion of the association between ACEs and smoking (21% for emotional abuse, 16% for physical abuse, 15% for physical neglect, 10% for parental separation or divorce). Among men, the associations between ACEs and smoking were not significant.

****Conclusions**:**

These findings suggest that for women, current smoking cessation strategies may benefit from understanding the potential role of childhood trauma.

## **Background**

Adverse childhood experiences (ACEs), which can include abuse (emotional, physical, and sexual), neglect (emotional and physical), and household dysfunction, are common among children [1]. In 2010 alone, maltreatment was reported for 695,000 US children (9.2 per 1000 children) [2]. In the largest study of ACEs, over 60% of 17,337 adults reported a history of at least one ACE [3].

Research suggests that ACEs have a long-term impact on the behavioral, emotional, and cognitive development of children [4-6]. This deleterious impact may be due to an unhealthy environment that impedes the resolution of early life development issues [7] as well as actual modifications in brain anatomy and functioning during important developmental periods [8]. These disruptions can lead to adoption of unhealthy coping behaviors throughout the lifespan [9,10] as well as maladaptive psychological functioning or psychological distress [11-14].

Persons who have experienced ACEs and psychological distress may smoke as a method to compensate for deficiencies in social and emotional development as well as a way to self-medicate biological dysregulations produced by abuse or neglect [5,15-18]. Smoking may be viewed as a viable coping option because of its perceived anxiolytic and sedative properties – for example, it’s ability to modify mood, manage dysphoria, regulate negative affect, control situational anxiety, and improve concentration [19-23]. As evidence, studies have shown that nicotine reduces anger in both smokers and nonsmokers with high hostility [24,25] and depressive symptoms in both nonsmokers and smokers with depression [26-28].

Psychiatric disorders are one of the most cited risk factors for nicotine dependence [29]. Longitudinal studies have suggested that depression [30-35], behavioral disorders [36], and anxiety [34], particularly PTSD [37], may increase the risk of subsequent smoking. Research has also implicated psychiatric conditions such as schizophrenia [38-41] and ADHD [42-44] as risk factors for smoking.

Several studies have examined the potential mediating effect of mental disorders on the relationship between ACEs and drug use. Studies conducted by Douglas et al. [45] and Lo and Chen [46] suggest that the relationship between childhood abuse and substance dependence may be partially mediated by mood and anxiety disorders. DeWit et al. [47], implicate social phobia as the mediator between adverse life events and chronic stress in childhood and drug dependence in adulthood. According to a literature review conducted by Simpson and Miller [48], psychiatric conditions such as depression and anxiety disorders mediate the relationship between child abuse and substance use disorders in women. Moreover, in a study conducted by White and Widom [49], the authors concluded that PTSD among maltreated girls may increase the risk of subsequent substance use problems.

Despite the fact that smoking continues to be the leading cause of death and disability in the United States [50], the magnitude and complexity of the relationship between ACEs and smoking is only beginning to be understood. Research conducted thus far suggests that ACEs are significantly associated with early smoking initiation, smoking maintenance, heavy smoking, and lifetime smoking across birth cohorts [51-56].

Because of the pervasive effect of ACEs throughout the life course and the deleterious effect of smoking on health, we sought to examine the potential mediating effect of psychological distress on the relationship between ACEs and current adult smoking. Moreover, as current research suggests that child abuse and neglect may affect men and women differently [57] and that stressors leading to smoking initiation and maintenance may vary by sex [58-62], the relationships between ACEs and smoking were further explored by sex. The purpose of this study was threefold: 1. to examine the relationships between ACEs, psychological distress, and adult smoking; 2. to determine if there were sex differences in the relationships between ACEs, psychological distress, and adult smoking, and 3. to determine if psychological distress mediated the relationship between ACEs and adult smoking among males and females.

## **Methods**

### **Study setting and participants**

The ACE Study is one of the largest studies to examine childhood trauma as a precursor of adult health in a managed care sample [63]. Data for the current study are based upon Wave II of the ACE Study, which were collected between April and October of 1997. These data were used to examine the relationship between multiple categories of childhood trauma (ACEs) and health and behavioral outcomes later in life. Participants were drawn from adult members of the Kaiser Permanente Medical Care Program in San Diego, California, undergoing a free comprehensive medical examination through the Health Appraisal Clinic (HAC), Department of Preventive Medicine [64].

A total of 13,330 Kaiser Health Plan members completed standardized medical evaluations at the HAC from April through October of 1997. Questionnaires were completed by 8,667 San Diego, California, Kaiser Permanente Health Maintenance Organization members who agreed to participate in the survey. Among these, 7,210 (83.2%) respondents completed information for the study variables and were included in the analyses (3,895 females and 3,315 males).

### **Survey methods and variable definitions**

Prior to the medical examination at the clinic, each Kaiser member attending the San Diego HAC completed a standardized health appraisal questionnaire and the Standard Form-36 (SF-36) questionnaire, which was used to assess functional health and well-being [65,66]. After the physical exam, patients were mailed the study’s Family Health History (FHH), a 168-item questionnaire that covers a broad range of childhood exposures and current health behaviors. Participation was voluntary, and patients were assured that the FHH would not become part of their medical record.

*Adverse childhood experiences* were defined using items from the FHH. The following ten ACE categories were assessed: emotional abuse (2 questions), physical abuse (2 questions), sexual abuse (4 questions), emotional neglect (5 questions), physical neglect (5 questions), witnessing domestic violence against mother or stepmother (4 questions), alcoholic or drug-abusing family members (2 questions), mentally ill household members (2 questions), parents separated or divorced, and incarcerated household members (1 question each) (Table 1). Verbatim ACE study questions can be found at: http://www.cdc.gov/ace/questionnaires.htm. Questions from published surveys were used to construct these ACE items. Questions adapted from the Conflicts Tactics Scale were used to define psychological and physical abuse during childhood and to define violence against the respondent’s mother or stepmother [67]. Four questions adapted from the Wyatt Sexual History Questionnaire [68] were used to define sexual abuse during childhood. Questions about exposure to alcohol or drug abuse during childhood were taken from the 1988 National Health Interview Survey [69]. Physical and emotional neglect were assessed by using the Childhood Trauma Questionnaire short form [70].

**Table 1:** Definitions of abuse, neglect, and household dysfunction that occurred before age 19 years

**Category**	**Definitions**
Abuse	
Emotional	At least one of the following responses:
	1. Often or very often a parent or other adult in the household swore at you, insulted you, or put you down.
	2. Sometimes, often, or very often they acted in a way that made you think that you might be physically hurt.
Physical	At least one of the following responses:
	1. Sometimes, often, or very often you were pushed, grabbed, slapped, or had something thrown at you.
	2. Sometimes, often, or very often hit so hard that you had marks or were injured.
Sexual	At least one affirmative (yes) response about an adult or a person at least 5 years older:
	1. Ever touched or fondled you in a sexual way.
	2. Had you touch their body in a sexual way.
	3. Attempted oral, anal, or vaginal intercourse with you.
	4. Actually had oral, anal, or vaginal intercourse with you.
Neglect	
Emotional	5 Childhood Trauma Questionnaire (CTQ) questions (Bernstein, et al., 1994) had possible responses of “never true’, “rarely true”, “sometimes true”, “often true”, or “very often true”. Responses were reverse scored on a Likert scale ranging from 5 to 1, respectively.
	1. There is someone in my family who helped me feel important or special.
	2. I felt loved.
	3. People in my family looked out for each other.
	4. People in my family felt close to each other.
	5. My family was a source of strength and support.
	A total cumulative score of 15 and higher (moderate to extreme on the CTQ clinical scale) defined childhood emotional neglect (Bernstein, et al., 1994).
Physical	5 Childhood Trauma Questionnaire (CTQ) questions (Bernstein, et al., 1994) had possible responses of “never true’, “rarely true”, “sometimes true”, “often true”, or “very often true”. Responses were scored on a Likert scale ranging from 1 to 5, respectively with items 2 and 5 reverse scored (5 to 1, respectively). :
	1. You did not get enough to eat.
	2. You knew there was someone to take care of you and protect you.
	3. Your parents were too drunk or high to take care of the family.
	4. You had to wear dirty clothes.
	5. There was someone to take you to the doctor if you needed it.
	A total cumulative score of 10 or higher (moderate to extreme on the CTQ clinical scale) defined childhood physical neglect (Bernstein, et al., 1994).
Household dysfunction	
Witnessing domestic violence	At least one affirmative (yes) response to the following about your mother or stepmother:
	1. Sometimes, often, or very often was pushed, grabbed, slapped, or had something thrown at her.
	2. Sometimes, often, or very often was kicked, bitten, hit with a fist, or hit with something hard.
	3. Was ever repeatedly hit over at least a few minutes.
	4. Was ever threatened or hurt by a knife or gun.
Household substance abuse	At least one affirmative (yes) response about living with anyone (before age 18) who:
	1. Was a problem drinker or alcoholic.
	2. Used street drugs.
Household mental illness	At least one affirmative (yes) response about a household member who:
	1. Was depressed or mentally ill.
	2. Attempted suicide.
Parental separation or divorce	Parents were ever separated or divorced.
Incarcerated household member	A household member went to prison.

In addition to examining individual ACEs, an *ACE score* was constructed to examine the cumulative exposure to the different types of abuse, neglect, and household dysfunction [3,52,64,71]. Exposure to any ACE counted as one point, and categories were summed for a total score between 0 and 10 points. The ACE score summarizes the total number of ACEs an adult recalls experiencing as a child or adolescent across the ten categories.

*Psychological distress* was assessed as a continuous variable and used the Mental Component Summary (MCS) score calculated from the SF-36. The SF-36 is a generic, multipurpose, short-form health survey with 36 questions and eight subscales [66,72]. The eight scales (Physical Functioning, Role Physical, Bodily Pain, General Health, Vitality, Social Functioning, Role Emotional, and Mental Health) form two distinct higher-ordered clusters, designated physical health (PCS) and mental health (MCS), which account for 80%–85% of the variance in the eight scales [73,74]. The reliability estimates of the two summary scales have generally exceeded 0.90 [65]. Predictive studies of validity have linked SF-36 scale scores and the MCS score to the clinical course of depression [75-78]. The MCS score is calculated from a complex set of computer-generated algorithms. All eight scales comprise the MCS score, but three scales (Mental Health, Role Emotion, and Social Functioning) correlate most highly and contribute most to the scoring [65]. Role Physical, Bodily Pain, Vitality, Social Functioning, Role Emotional, and Mental Health all reference the past four weeks. Physical Functioning and General Health reference the respondent’s current health and functional status [65]. As the mean MCS score decreases, level of psychological distress increases. The general US population mean MCS norm score for males is 50.73 and for females is 49.33 [65].

*Current smoking status* was assessed using two questions: a) “Have you smoked at least 100 cigarettes in your entire life?, and b) “Do you smoke cigarettes now?” Persons who had smoked at least 100 cigarettes in their lifetime and smoked at the time of the survey were considered adult current smokers [52,79-81].

### **Statistical analysis**

Mediation analyses were conducted to identify and explain the relationship between ACEs and adult current smoking based on the inclusion of the MCS score as an indicator for psychological distress [82]. In logistic models that included psychological distress and ACEs (individual or total score) as independent variables and adult current smoking as the dependent variable, psychological distress was treated as a potential mediating variable [82,83].

**Figure 1:**
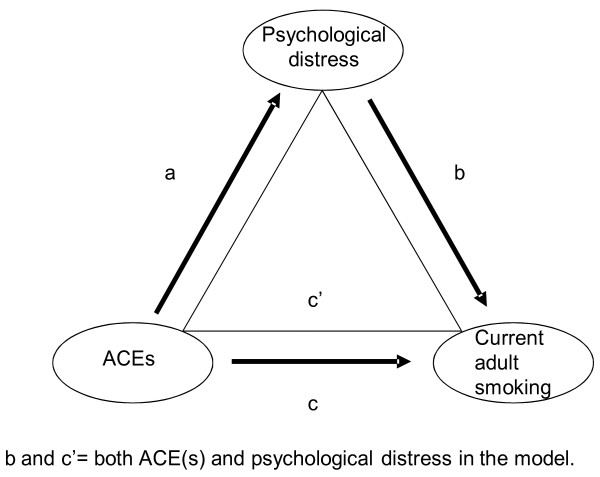
Mediation model.

Several criteria must be satisfied in order for mediation analysis to be valid (Figure 1). First, the independent variable (ACEs) must be significantly associated with the dependent variable (adult current smoking) (c coefficient); the mediating variable (psychological distress) must be significantly associated with the dependent variable (adult current smoking) with the independent variable (ACEs) included in the model (b coefficient); and the independent variable (ACEs) must be significantly associated with the mediating variable (psychological distress) (a coefficient) [84]. Second, the independent variable (ACEs) must be known to cause the mediation variable (psychological distress), which in turn causes the dependent variable (adult current smoking) [84].

The Sobel test was used to determine the significance of the indirect effect of the independent variable (ACEs) on the dependent variable (adult current smoking) through the mediator (psychological distress) [85]. Because the dependent variable (adult current smoking) and the independent variable (ACEs) were dichotomous and the mediating variable (psychological distress) was continuous, the coefficients in the mediation analyses were on two different scales. To make the coefficients compatible, we used techniques developed by MacKinnon and Dwyer [86] to calculate the Sobel statistic.

All models were first examined without being adjusted and were then adjusted for age group (18–34, 35–54, 55–74, and 75 years or older), race/ethnicity (white, black, Hispanic, Asian, Native American, and other), education (no high school diploma, high school or general educational development, some college/technical school, and college graduate), parental smoking during childhood (yes vs. no) to control for familial/genetic tendencies to smoke, and alcohol use in the previous month (yes vs. no) given that alcohol and smoking are highly correlated [87]. All statistical analyses were conducted using SAS 9.2 and Excel. Significance was tested at an alpha level of *p* < .05.

## **Results**

### **Descriptive characteristics**

The sample consisted of 3,895 females (54.0%) and 3,315 males (46.0%). The mean age was 55.9 years; nearly three-quarters of the participants were white and had at least some college or technical education (Table 2). Because there were different sex by effect interactions in the three primary mediation models, all analyses were stratified by sex.

**Table 2:** Selected characteristics of the population by sex

**Characteristics**	**Females (n = 3,895)**		**Males (n = 3,315)**		**Chi-square test**	
	**n %**		**n %**		**Value p-value (df)**	
**Age group (years)**						
18-34	440	11.3	234	7.1	52.7 (3)	<0.0001
35-54	1516	38.9	1213	36.6		
55-74	1563	40.1	1481	44.7		
75+	376	9.7	387	11.7		
**Mean age (SD)**		54.8 (15.4)		57.3 (14.4)		
**Race**						
White	2874	73.8	2517	75.9	19.3 (5)	0.0017
Black	158	4.1	132	4.0		
Hispanic	428	11.0	340	10.3		
Asian	353	9.1	226	6.8		
Native American	13	0.3	14	0.4		
Other	69	1.8	86	2.6		
**Education**						
No high school diploma	306	7.9	214	6.5	88.7 (3)	<0.0001
High school/GED	653	16.8	402	12.1		
Some college/technical	1666	42.8	1280	38.6		
College graduate	1270	32.6	1419	42.8		
**Alcohol consumed in past month**	2172	55.8	2158	65.1	65.0 (1)	<0.0001
**History of parental smoking**	2791	71.7	2434	73.4	2.8 (1)	0.0940
Mean MCS score (SD)*		51.2 (9.5)		53.2 (8.2)		
**Current smoker**	294	7.6	281	8.5	2.1 (1)	0.1470

*Mental Component Summary Score (MCS) based on SF-36.

Approximately 56% of women consumed alcohol in the previous month prior to the survey as did 65% of men. Over 70% of men and women reported that one or more parent smoked during their childhood. Approximately 7.6% of the women in the survey currently smoked as did 8.5% of men. The MCS score was slightly lower for women than men (51.2 versus 53.2).

Women were significantly more likely than men to report childhood emotional abuse (11.7% versus 8.2%), sexual abuse (24.2% versus 16.7%), emotional neglect (16.4% versus 12.2%), parental separation or divorce (25.3% versus 22.4%), mental illness in the household (25.0% versus 14.6%), household substance abuse (29.9% versus 25.5%), and an incarcerated household member (6.9% versus 4.8%) (Table 3). Men were significantly more likely than women to report physical abuse (28.6% versus 24.6%) and neglect (10.5% versus 8.6%).

**Table 3:** ACE characteristics of study sample by sex

**ACE**	**Females (n = 3,895)**	**Males (n = 3,315)**	**Chi-square test**	
	**%**	**%**	**Value (df)**	**p-value**
**Abuse**				
Emotional	11.7	8.2	23.8 (1)	<0.0001
Physical	24.6	28.6	14.5 (1)	0.0001
Sexual	24.2	16.7	61.2 (1)	<0.0001
**Neglect**				
Emotional	16.4	12.2	25.2 (1)	<0.0001
Physical	8.6	10.5	7.7 (1)	0.0054
**Household dysfunction**				
Witnessing domestic violence	13.6	12.1	3.8 (1)	0.0526
Parental separation or divorce	25.3	22.4	8.6 (1)	0.0034
Mental illness in household	25.0	14.6	119.4 (1)	<0.0001
Household substance abuse	29.9	25.5	17.4 (1)	<0.0001
Incarcerated household member	6.9	4.8	14.6 (1)	0.0001
**Total number of ACEs**				
0	32.1	34.7	50.2 (4)	<0.0001
1	24.1	26.9		
2	14.7	16.1		
3	10.4	9.3		
4+	18.7	13.0		

### **Relationship between ACEs and current adult smoking**

**Figure 2:**
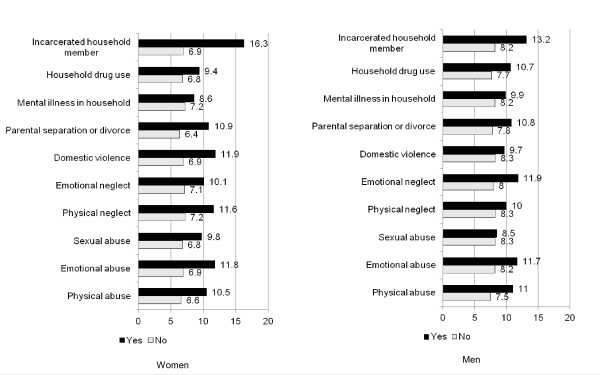
Prevalence of current smoking by ACE status and gender.

The unadjusted associations between current adult smoking and ACEs by sex can be found in Figure 2. After adjustment for sociodemographic characteristics, parental smoking during childhood, and alcohol use in the past month, the odds of adult smoking was at least 1.4 times greater among women who have been emotionally or physically abused, physically neglected, or had experienced parental separation or divorce (versus women who had not experienced each of these ACEs) (Table 4). Notably, the odds of smoking was markedly greater among women WHO had an incarcerated household member during childhood (AOR 2.3, 95% CI: 1.6-3.2) (versus women who had not had an incarcerated household member during childhood). While the odds of current adult smoking among women increased as the ACE score rose, this association was not significant after adjustment (Figure 1, c coefficient).

**Table 4:** Adjusted odds ratios (OR) and 95% confidence intervals for the relationships between ACEs and adult current smoking, by sex

**ACE**	**Females Adjusted**^**a**^**OR (95% CI)**	**Males Adjusted**^**a**^**OR (95% CI)**
**Abuse**		
Emotional		
Yes	1.4 (1.1-2.0)*	1.2 (0.8-1.8)
No	Referent	Referent
Physical		
Yes	1.4 (1.1-1.8)*	1.3 (1.0-1.7)
No	Referent	Referent
Sexual		
Yes	1.2 (0.9-1.6)	0.9 (0.7-1.3)
No	referent	Referent
**Neglect**		
Emotional		
Yes	1.2 (0.9-1.7)	1.2 (0.9-1.8)
No	Referent	Referent
Physical		
Yes	1.5 (1.1-2.2)*	1.1 (0.7-1.6)
No	Referent	Referent
**Household dysfunction**		
Witnessing domestic violence		
Yes	1.4 (1.0-1.9)	0.9 (0.6-1.3)
No	Referent	Referent
Parental separation or divorce		
Yes	1.4 (1.1-1.9)*	1.1 (0.8-1.5)
No	Referent	Referent
Mental illness in the household		
Yes	1.1 (0.8-1.4)	1.1 (0.8-1.5)
No	Referent	Referent
Household substance abuse		
Yes	1.0 (0.8-1.3)	1.1 (0.8-1.4)
No	referent	Referent
Incarcerated household member		
Yes	2.3 (1.6-3.2)*	1.1 (0.7-1.8)
No	Referent	Referent
**ACE score**		
0	Referent	Referent
1	0.7 (0.5-1.0)	1.2 (0.8-1.7)
2	1.3 (0.9-1.8)	1.3 (0.9-2.0)
3	1.3 (0.9-2.0)	0.9 (0.6-1.5)
4+	1.4 (1.0-2.0)	1.2 (0.8-1.7)

^a^ Odds ratios (OR) and 95% confidence intervals (CI). Multivariable logistic regression models included age group, race, education, parental smoking during childhood, and alcohol use in past month.

*p < 0.05.

Among men, the prevalence and unadjusted odds of current adult smoking was significant for physical abuse, emotional neglect, parental separation or divorce, living with a family member who abused substances, and having an incarcerated household member. Notably, after adjusting for covariates, none of these associations were significant.

### **Relationship between ACEs and psychological distress**

**Figure 3:**
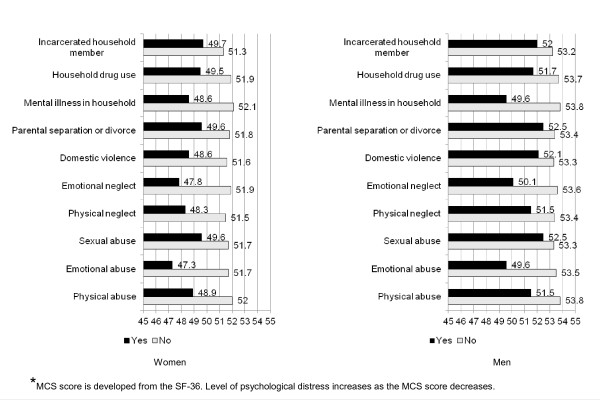
Mean Mental Component Summary (MCS) score by ACE status and gender.

In the unadjusted models, the mean MCS score, an indicator of psychological distress, was lower among those with any ACE compared to those without the given ACE among both women and men (with the exception of incarcerated household member among men), suggesting increased psychological distress (Figure 3). In the adjusted linear regression models among women (Table 5), all associations between the individual ACEs and the psychological distress indicator were significant except for that in which the independent variable was a childhood exposure to incarcerated household members. Among men, all adjusted associations with the psychological distress indicator were significant except for those in which the independent variable was parental separation or divorce or incarcerated household member. As the ACE score increased in both adjusted and unadjusted linear regression models, the level of psychological distress also increased (i.e., mean score decreased as number of ACEs increased) (Figure 1, a coefficient).

**Table 5:** **Adjusted coefficients**^**a**^**and 95% confidence intervals for the relationships between ACEs and psychological distress, by sex**

**ACE**	**Females Adjusted**^**a**^**Coefficients (95% CI)**	**Males Adjusted**^**a**^**coefficients (95% CI)**
**Abuse**		
Emotional	−3.9 (−4.8- -3.0)*	−3.6 (−4.6- -2.6)*
Physical	−2.6 (−3.3- -1.9)*	−2.1 (−2.7- -1.5)*
Sexual	−1.8 (−2.5- -1.1)*	−0.9 (−1.6- -0.1)*
**Neglect**		
Emotional	−3.7 (−4.5- -2.9)*	−3.4 (−4.2- -2.5)*
Physical	−2.8 (−3.8- -1.7)*	−1.9 (−2.7- -1.0)*
**Household dysfunction**		
Witnessing domestic violence	−2.4 (−3.2- -1.5)*	−0.9 (−1.7- -0.01)*
Parental separation or divorce	−1.6 (−2.3- -0.9)*	−0.6 (−1.3-0.03)
Mental illness in the household	−3.1 (−3.8- -2.5)*	−4.0 (−4.8- -3.3)*
Household substance abuse	−1.7 (−2.4- -1.0)*	−1.7 (−2.3- -1.1)*
Incarcerated household member	−0.9 (−2.1-0.2)	−0.9 (−2.2-0.4)
**ACE score**	−1.1 (−1.3- -0.9)*	−1.0 (−1.2- -0.8)*

^a^Multivariable linear regression models included age group, race, education, parental smoking during childhood, and alcohol use in past month.

*p < 0.05.

### **Relationship between psychological distress and current adult smoking**

In the models adjusted for sociodemographic characteristics, parental smoking during childhood, and past 30 day consumption of alcohol, the association between psychological distress and current adult smoking was significant for both women (AOR = 0.98; 95% CI: 0.97-0.99; Wald chi-square = 15.22, DF = 1, p-value <0.0001) and men (AOR = 0.98; 95% CI: 0.97-1.00; Wald chi-square = 5.73, DF = 1, p = 0.0166). With the addition of each ACE in the model, the associations remained significant for women (AOR = 0.98; 95% CI: 0.97-0.99; p-value range: p = 0.0004 for model with mental illness in household to p = 0.0014 for model with emotional abuse) but not men (AOR = 0.99, 95% CI: 0.87-1.00, p-value range: p = 0.0523 for the model with sexual abuse to p = 0.0934 for the model with incarcerated household member) (Figure 1, b coefficient).

### **Mediating role of psychological distress on the relationship between ACEs and current adult smoking**

Given that after adjusting for covariates, there was no significant association between any of the ACEs and smoking or psychological distress and smoking (with ACEs included) among men, mediation analyses were limited to women. After adjusting for covariates, psychological distress mediated 22% of the relationship between emotional abuse and current adult smoking, approximately 17% of the relationship between physical abuse and current adult smoking, 14% of the relationship between physical neglect and current adult smoking, and about 10% of the relationship between parental separation or divorce and current adult smoking among women (Table 6) (Figure 1, c’ coefficient).

**Table 6:** Sobel statistics and percent mediated. Women

	**Sobel Test**^**a**^		**% mediated**^**b**^
**ACE**	Test Statistic (SE)	p-value	
**Abuse**			
Emotional	−3.36 (0.02)	0.0008	22.0%
Physical	−3.29 (0.02)	0.0010	16.8%
**Neglect**			
Physical	−3.01 (0.02)	0.0026	14.0%
**Household dysfunction**			
Parental sep/div	−2.88 (0.01)	0.0040	10.2%

*Note.* Sobel test and proportion of mediation obtained from linear and logistic regression models adjusted for covariates including age group, race, education, parental smoking during childhood, and alcohol use in past month; P-values drawn from the normal distribution under the assumption of a 2-tailed z-test. The hypothesis is that the mediated effect equals zero.

Excel spreadsheet created by Nathaniel R. Herr (February, 2006), Adopted from Kenny, 2006. Available at: http://nrherr.bol.ucla.edu/Mediation/logmed.html.

^a^Sobel test. http://www.quantpsy.org/sobel/sobel.htm

^b^equation (c-c’)/c.

## **Discussion**

This research reveals several important findings. First, there are differences in the relationship between ACEs, psychological distress, and adult smoking by sex. While the relationships between ACEs and psychological distress was evident among both men and women, after adjusting for covariates, there were not significant relationships between ACEs and smoking or psychological distress and smoking (after each ACE was added to the model) among men. Second, this research suggests that women, particularly those who have experienced emotional or physical abuse, physical neglect, or parental separation or divorce as children may be at particular risk for smoking in adulthood. In fact, approximately 22% of the relationship between emotional abuse and adult smoking was mediated though psychological distress as was 17% of the relationship between physical abuse and adults smoking, 14% of the relationship between physical neglect and adult smoking, and 10% of the relationship between parental separation and divorce and adult smoking.

Our findings confirm results of earlier research suggesting sex differences in smoking behavior and pattern . Although negative affect, including depression, is related to smoking among both men and women, the relationship is much stronger for women [88-90]. In fact, recent research suggests that stressful childhood life events may disproportionately influence a women’s decision to use drugs [48-57]. This may be due in part to differences in coping styles and socialization [56]; females may develop more passive styles of responding to threats and distressing events as opposed to boys who may engage in a more active coping style [91-95]. Interestingly, women are often less dependent on nicotine then men [60,96-98], they are less likely to be heavy smokers [79], and have lower concentrations of cotinine (a byproduct of nicotine). Notably, however, studies have consistently found that women have lower quit rates than men [61,62,97], have lower confidence in their ability to quit [98,99], and often experience worse withdrawal symptoms during smoking cessation attempts [59,97]. In fact, recent research suggests that the smoking rates for adolescent and adult women may actually be increasing [100].

Our study indicates that women are more likely than men to report emotional and sexual abuse and emotional neglect while men are more likely to report physical abuse and neglect. Literature has consistently indicated that women are more likely than men to report sexual abuse [101] and men are more likely than women to report physical abuse [102]. The authors could find very little research that examined emotional abuse and neglect and physical neglect by sex; specifically research that did not contain the same data used in this study. The one study we did find indicated that women were significantly more likely than men to report emotional abuse and slightly more likely than men to report emotional neglect, although not statistically significantly so [103]. This same study indicated that men were significantly more likely than women to report physical neglect, results consistent with our findings [103].

Much research has already examined potential biases and limitations of the ACE Study data. Research conducted by Felitti et al. [64] determined that respondent and nonrespondent groups were similar with regard to sociodemographic characteristics (e.g., percentages of women, mean years of education, and marital status), self-rated health, engagement in adverse health behaviors (e.g., smoking and other substance abuse), and presence of chronic diseases such as heart attack, stroke, chronic obstructive lung disease, hypertension, and diabetes. Edwards et al. [104] conducted research examining potential response bias and found that persons who did not participate in the ACE Study experienced childhood sexual abuse at the same rate as those who agreed to participate; research made possible by a dichotomous screening question about childhood sexual abuse in the health history survey. Moreover, those who participated in the study who reported sexual abuse had similar levels of current mental and physical health problems as those who did not participate and also reported sexual abuse [104]. Test-retest reliability research conducted by Dube et al. [105], found that childhood sexual, physical, and emotional abuse, as well as forms of household dysfunction (i.e., mental illness in household, substance abuse in household, parental discord or divorce, incarcerated household member, and domestic violence), showed good Cohen’s Kappa agreement as defined by Fleiss [106] and Landis and Koch [107] (range = 0.46–0.86). Finally, while persons in the ACE Study are older, more educated, and less likely to smoke than the general population, ACE Study sexual and physical abuse estimates are similar to those derived from adult population-based surveys [108-110].

There are several limitation that warrants further examination. First, the ACE Study data are cross-sectional and do not collect specific information on temporality. Although most current literature suggests that the majority of psychiatric disorders associated with smoking occur prior to smoking initiation [29-44], other pathways have been posited (e.g., bidirectional association, common environmental and genetic factors for both, and smoking initiation prior to psychological distress) [111,112]. Notably, in a study designed to specifically examine the stress-smoking relationship among adolescents, negative life events and negative affect were related to an increase in smoking over time, with no evidence of reverse causation [35]. Additional longitudinal studies are needed to further clarify the relationships between ACEs, psychological distress, and smoking among adults. Second, there is undoubtedly more than one pathway that would lead an adolescent to smoking initiation (e.g., peer pressure). Moreover, there could be a cohort effect because participants in this study likely began to smoke at a time when smoking was more socially acceptable than it is now, and therefore the relative contribution of ACEs and psychological distress may increase or decrease as the rates of smoking decrease over time. Third, at the inception of the study, domestic violence was recognized to primarily occur against women. It is commonly known now that domestic violence occurs to both men and women in the household. Given this, our study has underestimated the prevalence of domestic violence in the household. Fourth, longitudinal follow-up studies of adults with documented childhood abuse suggested that retrospective reports of childhood abuse often underrepresented actual events [113-115]. However, in a recent study by Tourangeau and Yan [116], the authors indicate that respondents are less likely to underreport undesirable events and behaviors when the questions are self-administered and when the data are collected in private. Bias also may be introducted if there are differences in reporting retrospective information about childhood abuse by sex. In an article by Widom and Morris [114], among persons with a history of documented sexual abuse in childhood, fewer men than women later considered the event sexual abuse. Fifth, according to recent research, the joint effect of multiple ACEs on mental disorders are non-additive and often attenuate with age. This, combined with recall failure, often overestimates the effects of summary ACE scales [117]. Given this, as was found in this study, one might not expect to see a dose–response relationship between number of ACEs and psychological distress. Further research is needed to determine an appropriate summary measure for retrospective studies. Sixth, it is not plausible that women would have more exposure to several of the ACEs (eg, household dysfunction) than men. This suggests that women are more sensitive to several of the ACE measures or are more willing to report them. Finally, psychological distress is a non-specific concept that can encompass everything from temporary negative emotion to chronic mental disorders. However, research suggests that the MCS is a good predictor of depressive disorders [76].

This study has important policy implications for public health approaches to smoking cessation. Despite increasingly stronger disincentives to smoking, including higher tobacco taxes and fewer places to smoke, the rate of smoking in the U.S. fell only slightly, from 20.9% in 2005 to 19.3% in 2010. At this slow rate of decline, by 2020 the adult smoking rate will only have fallen to about 17% [118]. Given the strong association between ACEs and smoking, interventions targeted to trauma survivors may enhance the effectiveness of broader-based anti-smoking efforts.

## **Conclusions**

Several recent articles have suggested that persons who have experienced ACEs are more likely to smoke, but the exact mechanism linking ACEs with adult current smoking has not been fully elucidated. This research provides preliminary evidence that among women, psychological distress may be a potential intermediate variable in the relationship between ACEs and adult current smoking. As such, when addressing smoking cessation in clinical practice, it may be important to understand not only psychological distress, but the underlying role of childhood trauma. Having knowledge about childhood trauma history in clinical practice may provide the opportunity to integrate trauma focused interventions. Moreover, to create effective intervention and prevention programs, research should be conducted to further elucidate the causes, developmental paths, and critical points that link ACEs to smoking, especially in adolescence [57]. Identifying potential modifiable risk factors for smoking onset in adolescents (e.g., ACEs), as well as building resiliency and positive social support networks for abused children may decrease the prevalence of smoking among children and adolescents exposed to maltreatment. Research examining additional potential covariates, including temporality, intensity, frequency, and duration of maltreatment [119,120]; victim’s past and current environmental circumstances; and genetic influences on smoking behavior and mental illness is also warranted [121,122].

## **Competing interests**

The authors declare that they have no competing interests.

## **Authors’ contributions**

TWS developed the research question, conducted the statistical analysis, conducted the literature review, and wrote the initial draft of the manuscript.VJE, SRD and SD assisted with the statistical analysis. MW, AWP, SR, VJE, and JBC assisted with subject matter content. All authors read and approved the final manuscript.

## **Disclaimer**

The findings and conclusions in this article are those of the authors and do not necessarily represent the official position of the Centers for Disease Control and Prevention.
